# Supplementary material for: Structural insights into catalytic mechanism and product delivery of cyanobacterial acyl-acyl carrier protein reductase
Source: Nat Commun. 2020 Mar 23;11:1525. doi: 10.1038/s41467-020-15268-y (PMC7089970; doi:10.1038/s41467-020-15268-y)
Supplement: Supplementary file 1 — Supplementary Information [file 41467_2020_15268_MOESM1_ESM.pdf]

Supplementary Information

Structural insights into catalytic mechanism and product delivery of cyanobacterial acyl-acyl carrier protein reductase

Yu Gao, Hongmei Zhang, et al.

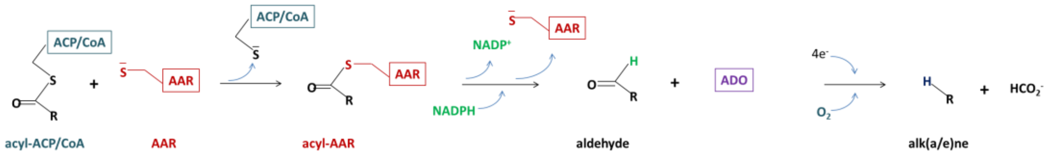

Supplementary Figure 1. Scheme of alk(a/e)ne biosynthesis pathway catalyzed by AAR and ADO

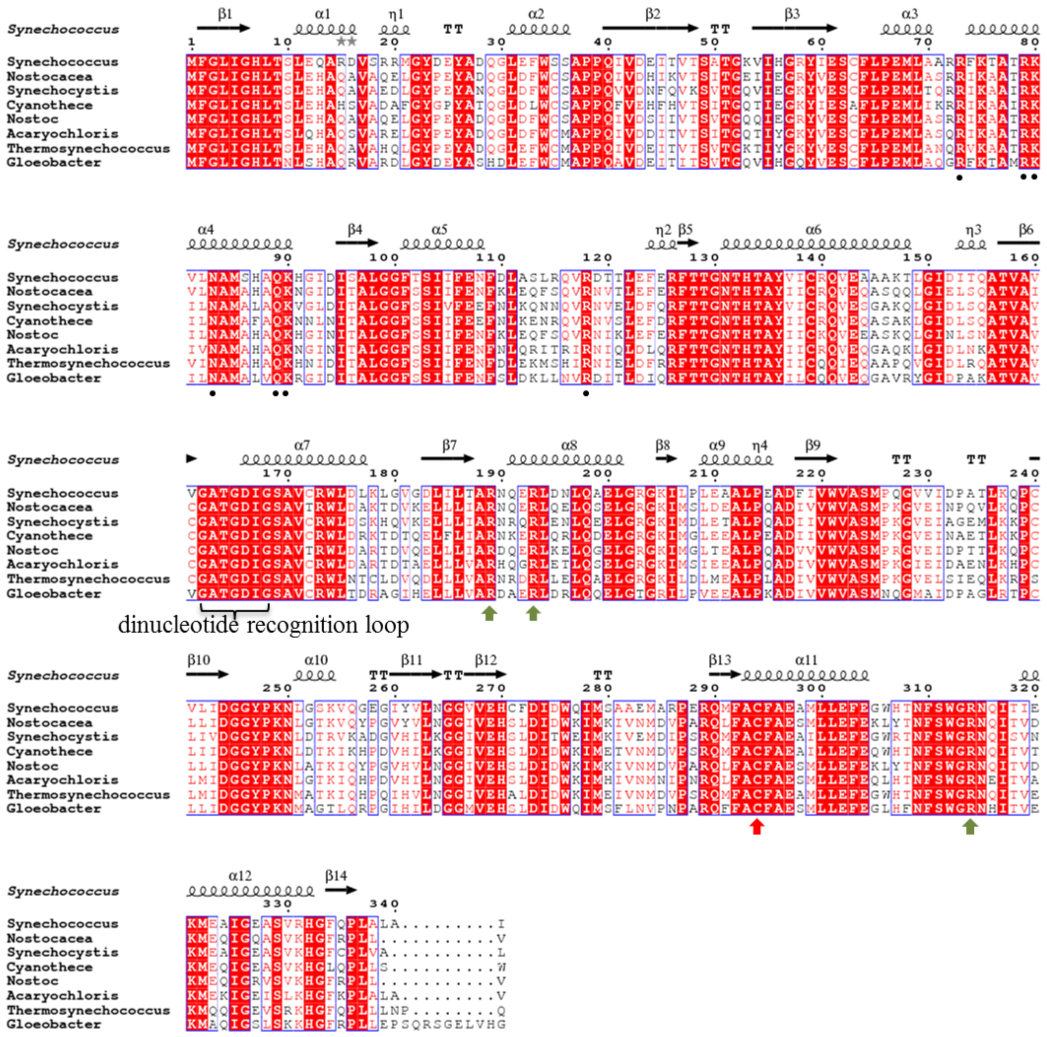

Supplementary Figure 2. Sequence alignment of AAR. Sequence alignment of *Se*AAR with other homologues. The sequences are from species: *Synechococcus elongatus* PCC7942, *Anabaena sp.* PCC7120 (*Nostocacea sp.* PCC7120), *Synechocystis sp.* PCC6803, *Cyanothece sp.* ATCC 51142, *Nostoc punctiforme* PCC 73102, *Acaryochloris marina*, *Thermosynechococcus elongatus* BP-1, *Gloeobacter violaceus* PCC7421. The secondary structure is displayed on top of *Se*AAR sequence. The dinucleotide recognition loop is indicated. The key residue C294 is indicated by a red arrow. The conserved residues involved in the interaction with ADO are labeled by black dots. The conserved residues R189, R193, R314 involved in NADPH binding are indicated by green arrows.

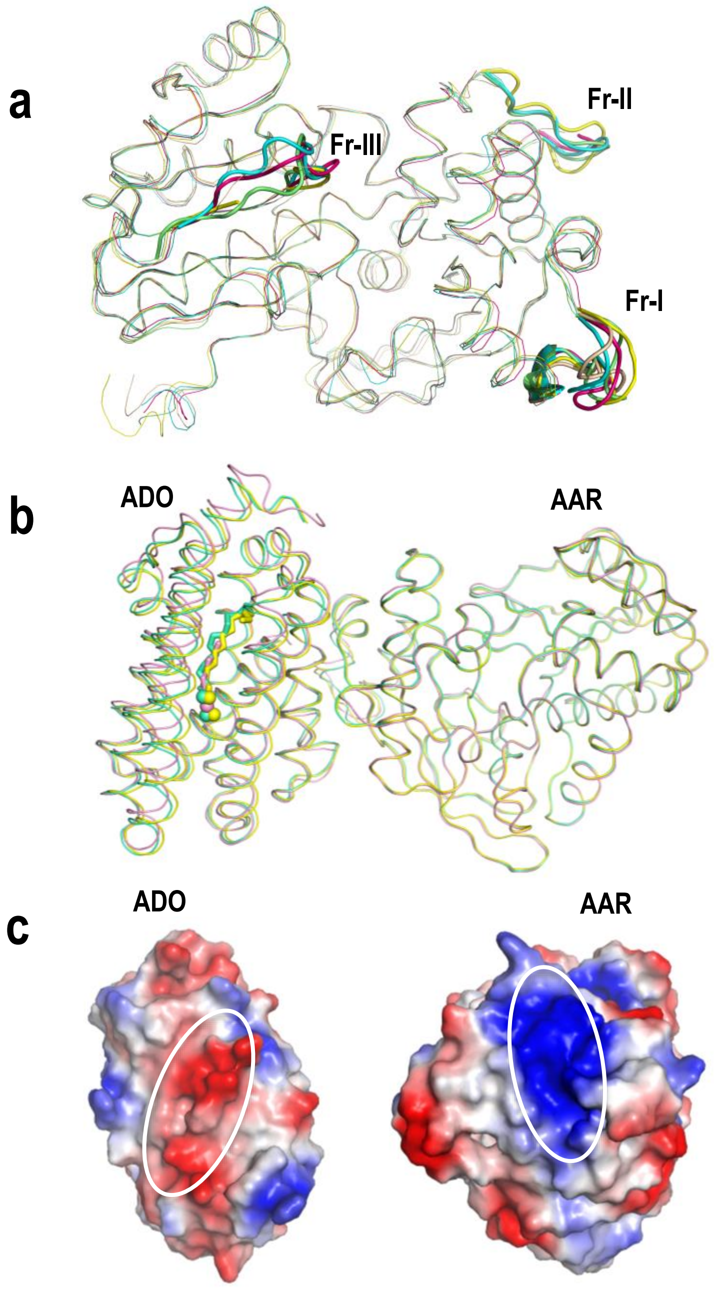

Supplementary Figure 3. Structure and intermolecular interaction of *Se*AAR and *Se*ADO. (a) Superposition of the five AAR molecules (shown in different color) in an asymmetric unit of AAR_{apo} structures. Three regions with evident conformational changes among the five molecules are labeled. **(b)** Superposition of three AAR-ADO complex structures. $AAR_{thio\text{-}ester}$-ADO, AAR_{NADPH}-ADO and $AAR_{stearoyl\text{-}CoA}$-ADO structures are shown in green, yellow and pink, respectively. The two iron atoms and a hydrocarbon chain bound with ADO are shown in spheres and sticks, respectively. The ligands bound with AAR are omitted for clarity. **(c)** Surface representations of AAR and ADO in the AAR complex. The positively charged region in the NTD of AAR and the negatively charged region of the helix 7 of ADO are involved in the intermolecular interaction and highlighted by white circles.

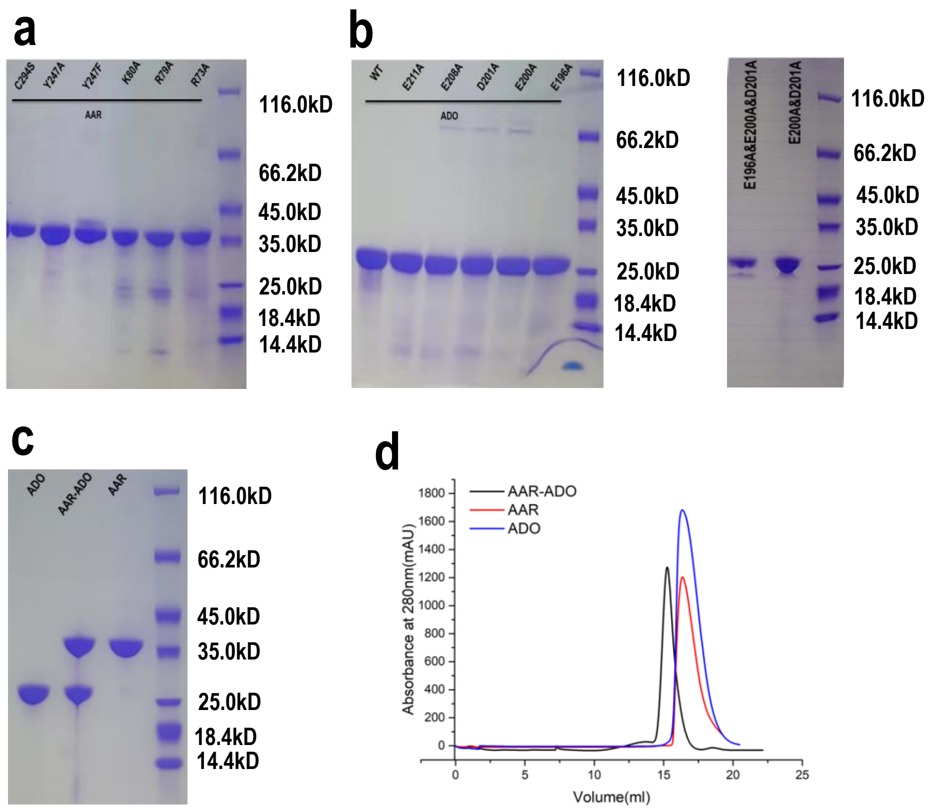

Supplementary Figure 4. Characterization of wild type (WT) and mutants of AAR and ADO proteins, and AAR-ADO complex. (a-c) The 4-20% gradient sodium dodecylsulfate polyacrylamide gel electrophoresis (SDS-PAGE) results of AAR wild type and its mutants **(a)**, ADO wild type and its mutants **(b)** and AAR-ADO complex **(c)**. **(d)** The size exclusion chromatography results of AAR (red), ADO (blue) and AAR-ADO complex (black). In each run, 500 uL of protein sample with concentration of 3mg·mL^{-1} was loaded on the Superdex 200 column (10/300, GE Healthcare). Source data of **(a-c)** are provided as a Source Data file.

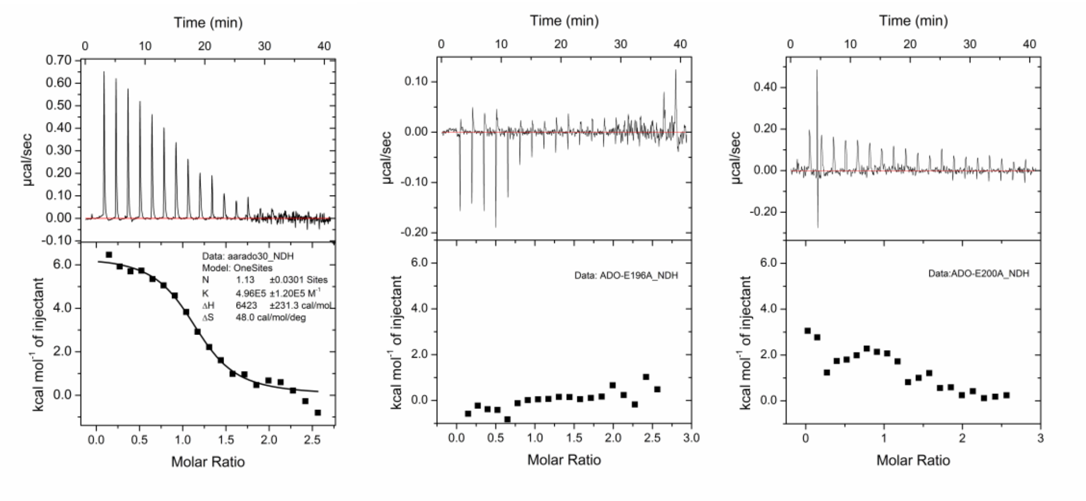

WT **E196A** **E200A**

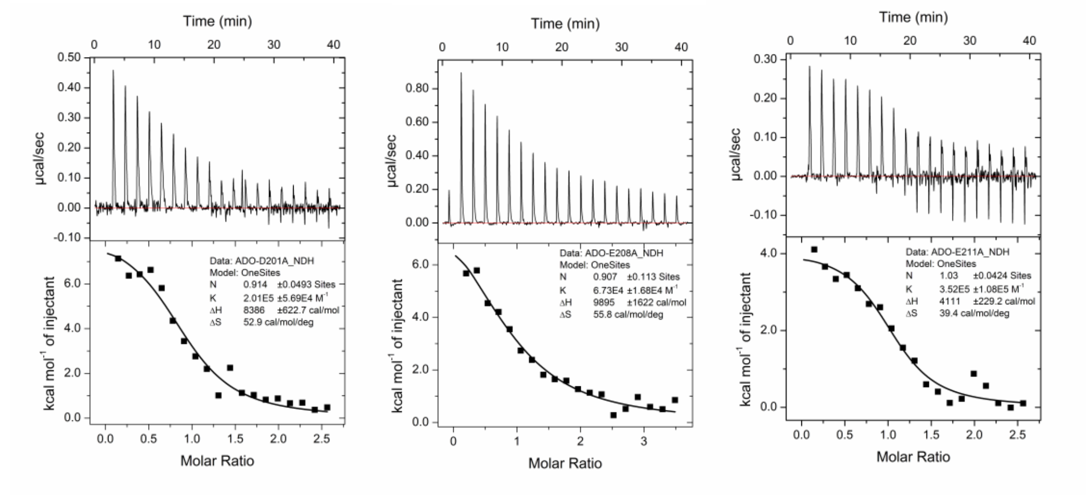

D201A **E208A** **E211A**

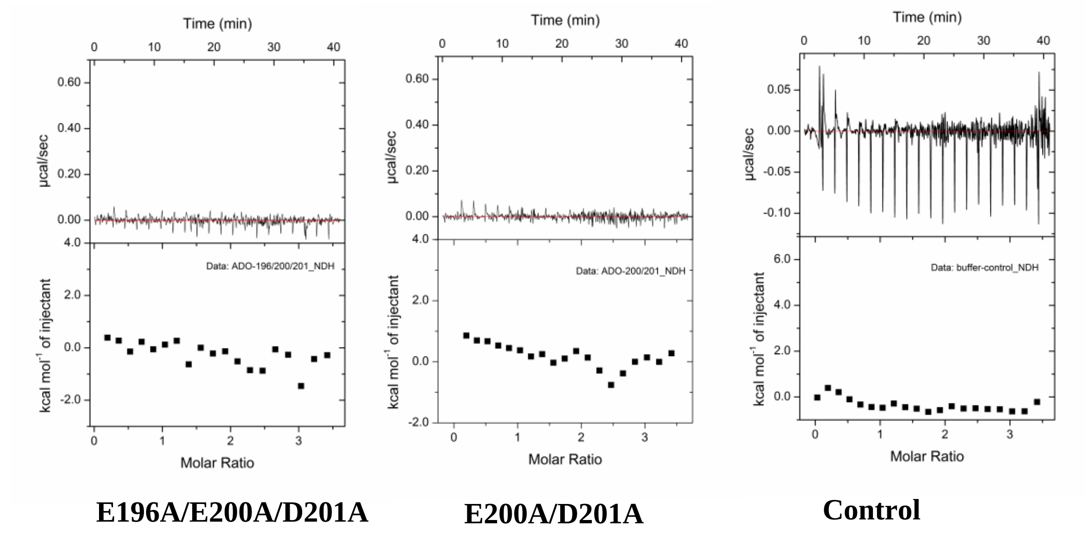

E196A/E200A/D201A **E200A/D201A** **Control**

Supplementary Figure 5. The binding assay of AAR with wild type (WT) and mutants of ADO measured by ITC.

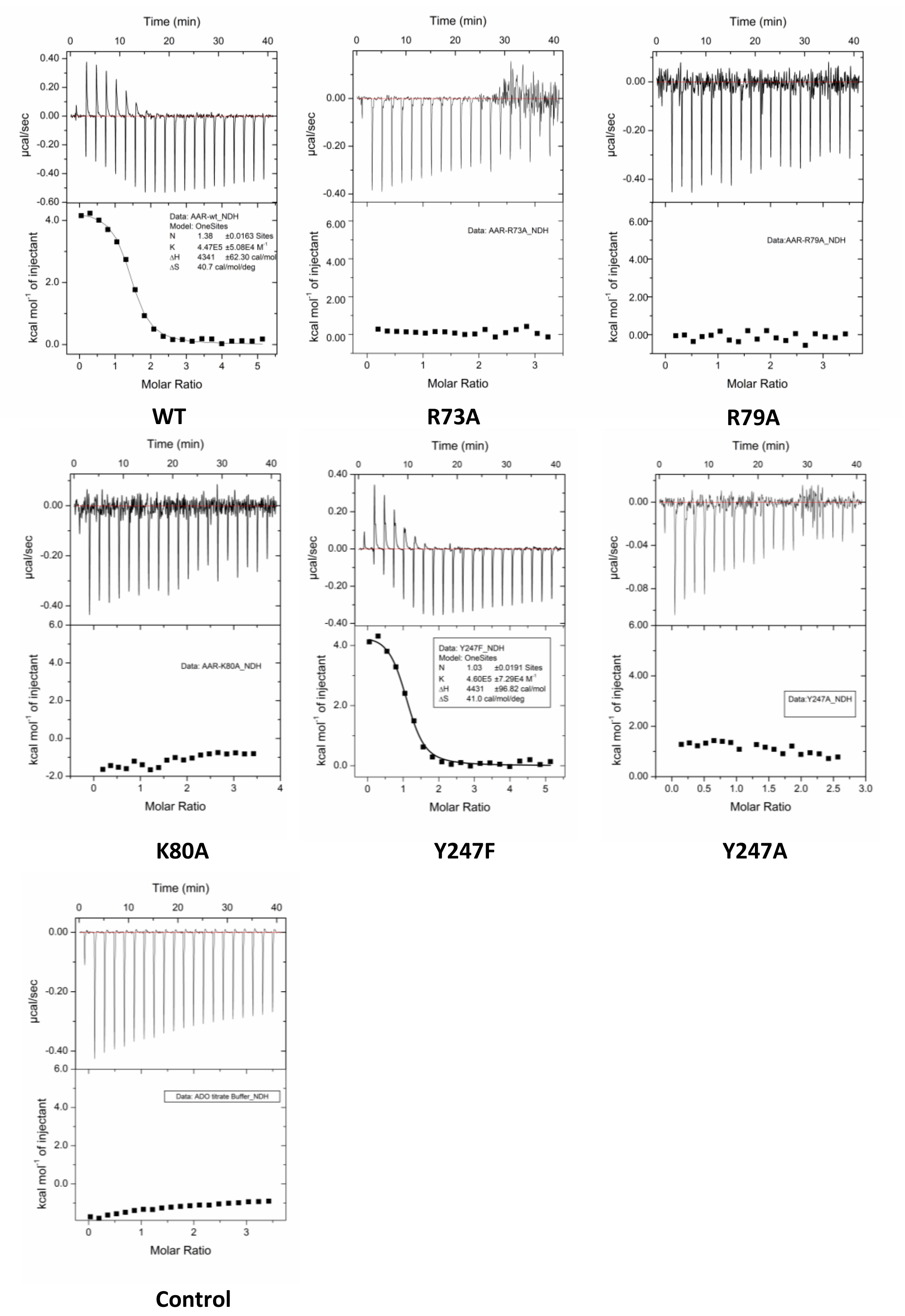

Supplementary Figure 6. The binding assay of ADO with wild type (WT) and mutants of AAR measured by ITC.

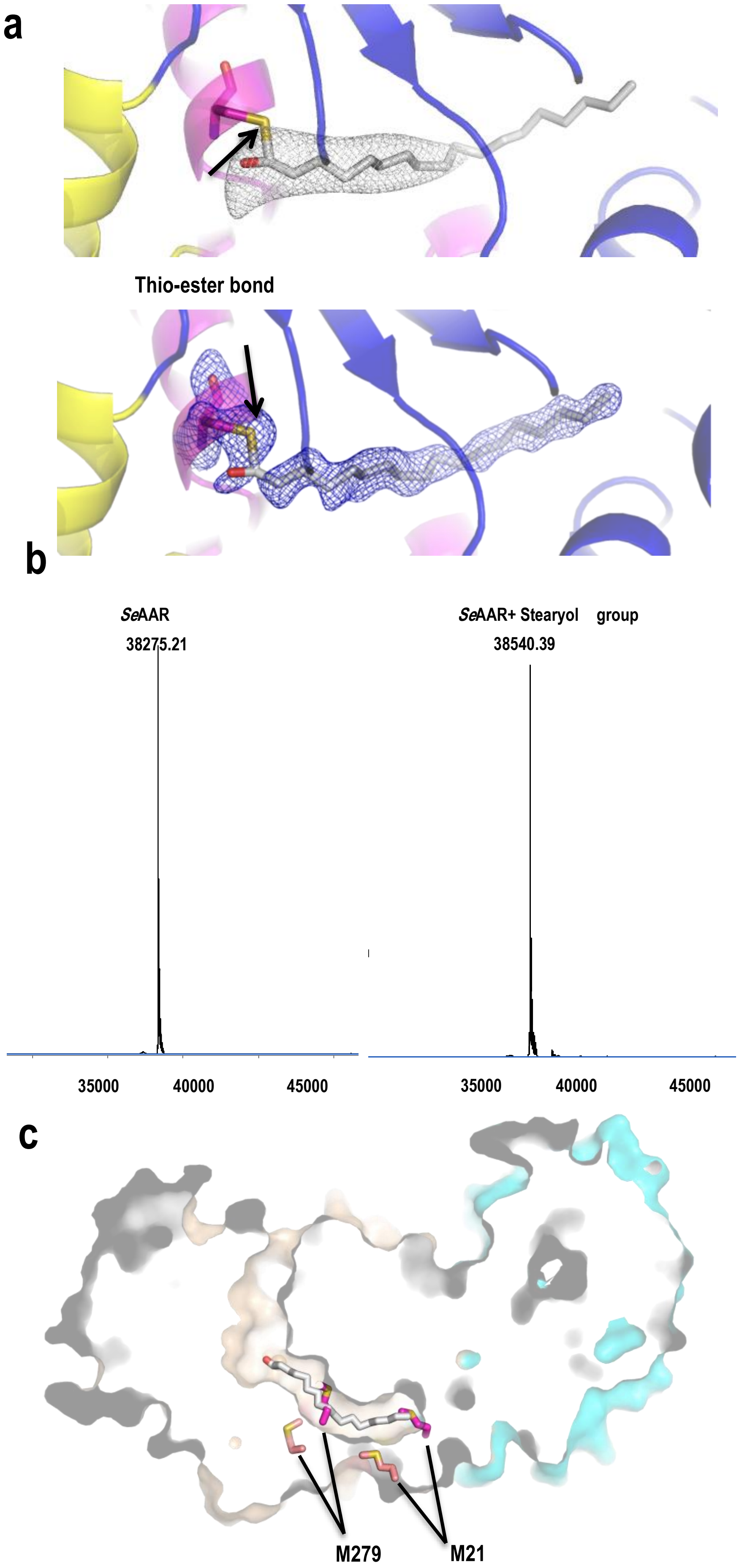
a
Thio-ester bond
b
SeAAR
38275.21
SeAAR+ Stearyol group
38540.39
35000
40000
45000
35000
40000
45000
c
M279
M21

Supplementary Figure 7. The AAR$_{thioester}$-ADO structure and the thio-ester bond. **(a)** The Fo-Fc (grey mesh, contoured at 3.5σ, upper panel) and 2Fo-Fc (blue mesh, contoured at 1σ, lower panel) electron density maps for stearoyl chain bound with C294 of AAR. The thio-ester bond formed between C294 and the stearoyl chain is indicated. **(b)** The mass spectrometric results of AAR alone (left panel) and the AAR sample incubated with stearoyl-CoA (right), which was used for crystallization. The theoretical molecular weights of *Se*AAR and the *Se*AAR covalently bound with stearoyl chain are 38273.82 Da and 38540 Da, respectively. **(c)** Superposition of the AAR part in AAR$_{thioester}$-ADO structure with AAR$_{apo}$ structure. AAR$_{thioester}$-ADO structure is shown in surface mode to highlight the hydrophobic tunnel in AAR, with AAR and ADO colored wheat and cyan, respectively. The stearic chain is shown as white stick. The two residues (M21 and M279) of AAR are shown in sticks and the carbon atoms are colored pink and magenta in AAR$_{thioester}$-ADO structure and AAR$_{apo}$ structure, respectively. These two residues undergo dramatic conformational changes between the two structures.

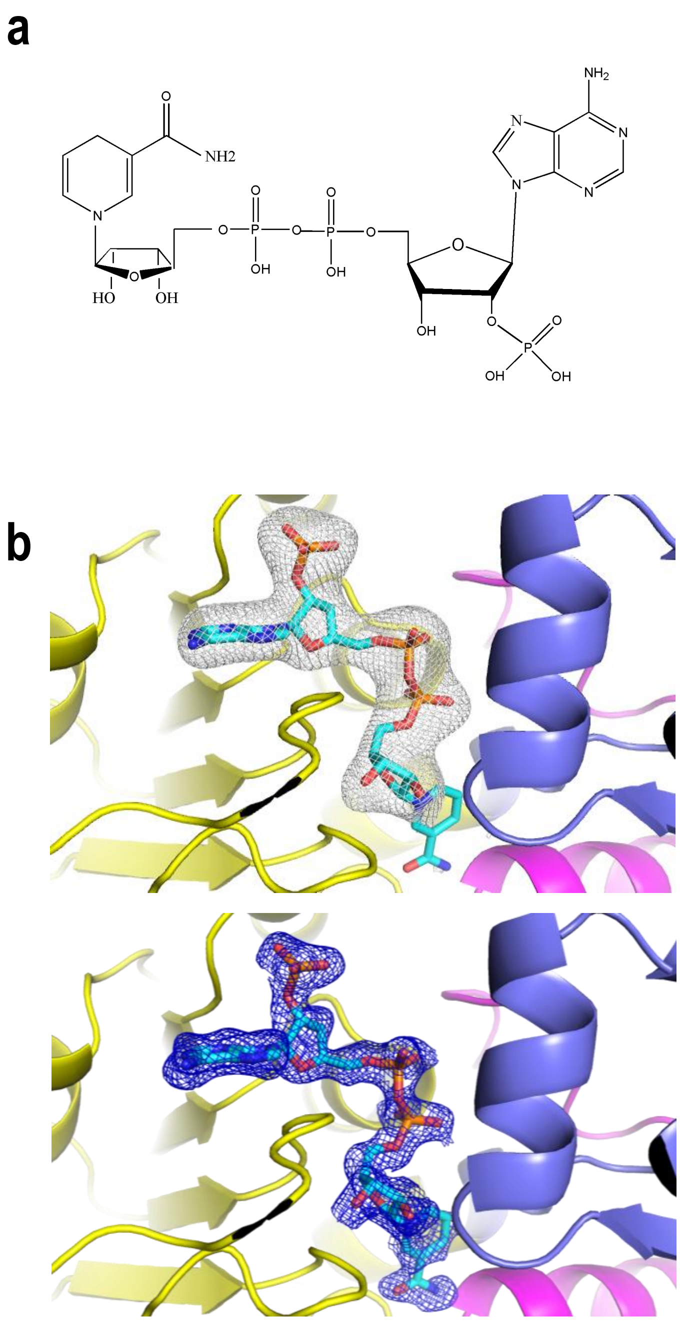

Supplementary Figure 8. **The NADPH bound in AAR_{NADPH}-ADO structure. (a)** Chemical structure of NADPH. **(b)** The Fo-Fc (grey mesh, contoured at 3.5σ, upper panel) and 2Fo-Fc (blue mesh, contoured at 1σ, lower panel) electron density maps of NADPH bound with AAR.

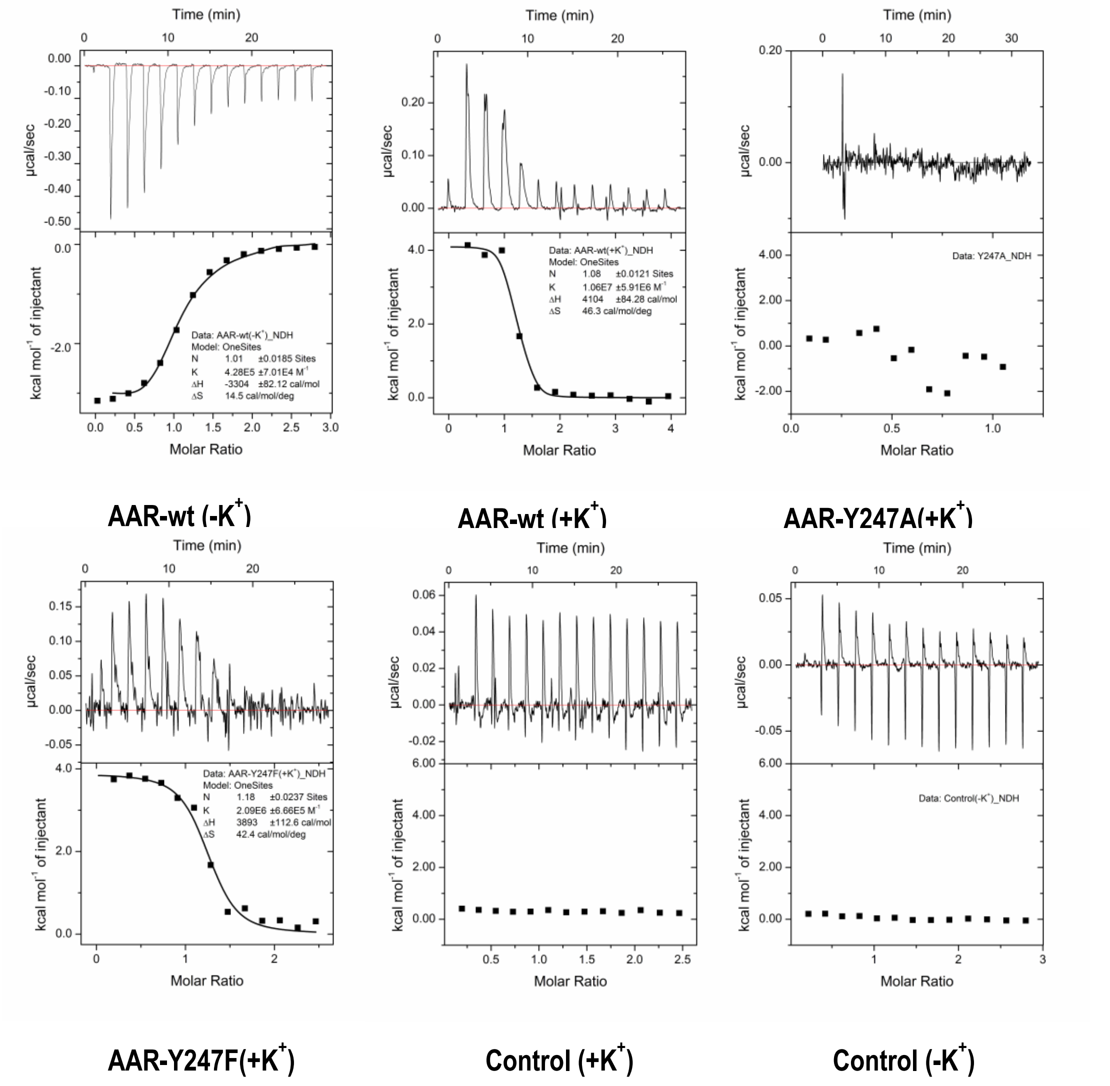

Supplementary Figure 9. The binding assay of wild type (wt) and mutants of AAR with the substrate stearoyl-CoA measured by ITC. During titration, K^+ ions are absent from the buffer (–K^+) or present in the buffer (+K^+).

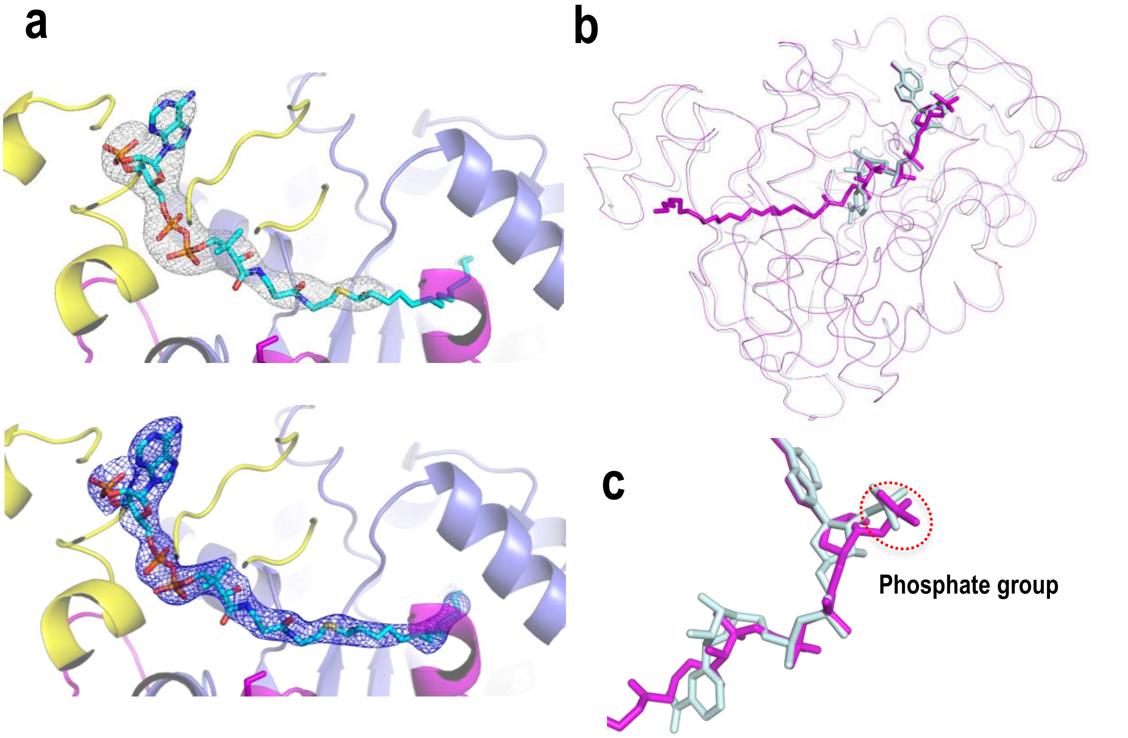

Supplementary Figure 10. The AAR$_{\text{stearoyl-CoA}}$-ADO structure. (a) The Fo-Fc (grey mesh, contoured at 3.5σ, upper panel) and 2Fo-Fc (blue mesh, contoured at 1σ, lower panel) electron density maps for stearoyl-CoA in AAR. **(b)** Superposition of AAR moiety in AAR$_{\text{NADPH}}$-ADO structure (light cyan) and AAR$_{\text{stearoyl-CoA}}$ structure (magenta) shown in ribbon mode. The stearoyl-CoA and NADPH shown as sticks occupy the same channel and are superimposed well. **(c)** Enlarged view of the superposition between stearoyl-CoA and NADPH. Both molecules have the same ADP moiety except for the different position of ribose phosphate group.

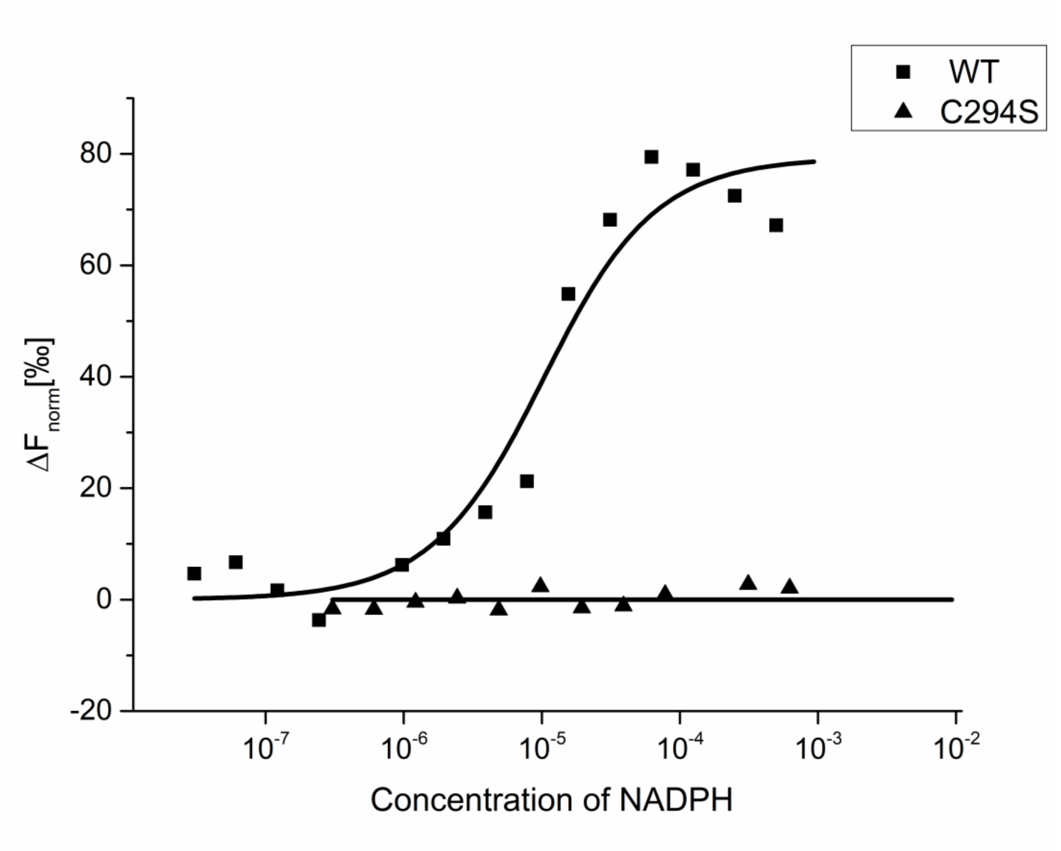

Name	Ligand Name	K_D(μM)
AAR-WT	NADPH	7.99
AAR-C294S	NADPH	N.D.

Supplementary Figure 11. The binding assay of AAR with NADPH measured by microscale thermophoresis (MST). The wild type AAR (AAR-WT) binds NADPH with a K_D value of 7.99 μM. The AAR C294S mutant incubated with stearoyl-CoA (AAR-C294S) is unable to bind NADPH, N.D. means not detected. Source data are provided as a Source Data file.

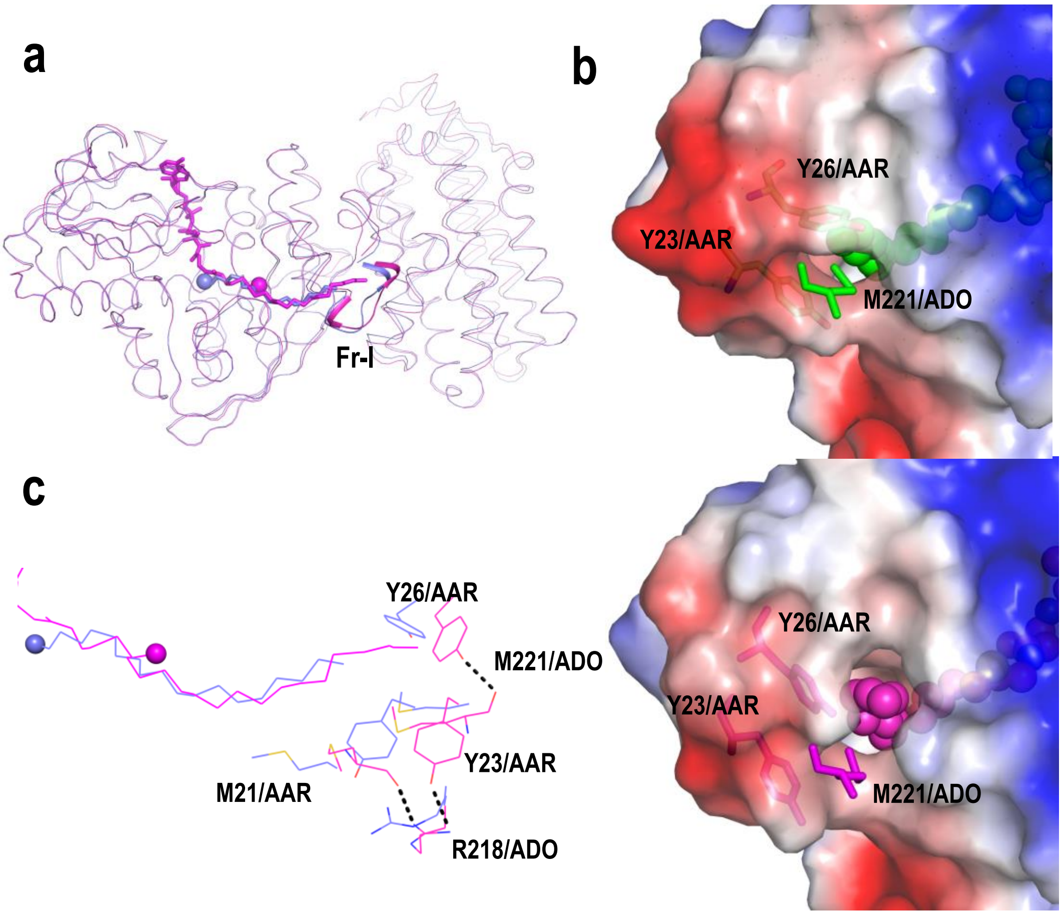

Supplementary Figure 12. Comparison of AAR$_{thioester}$-ADO structure and AAR$_{stearoyl\text{-}CoA}$-ADO structure. (a) Comparison of AAR$_{thioester}$-ADO structure (slate) with AAR$_{stearoyl\text{-}CoA}$-ADO structure (magenta) superposed on AAR part. The stearoyl-CoA and the stearic chain are shown in stick and the oxygen atoms linked to C1 atom are shown as spheres. The distance is about 6Å between the two oxygen atoms. A part of the Fr-I region (M21-Y26) of AAR in the two structures shows conformational change and is highlighted in cartoon mode and indicated. **(b)** The opening at the molecular surface of AAR in the AAR$_{thioester}$-ADO structure **(**upper panel**)** and AAR$_{stearoyl\text{-}CoA}$-ADO structure **(**lower panel**)**. The stearic chain is shown as spheres. Residues are shown as sticks. The bigger opening **(**lower panel) is formed at the molecular surface of AAR in AAR$_{stearoyl\text{-}CoA}$-ADO structure. **(c)** Structural comparison of the AAR-ADO interfacial region between AAR$_{thioester}$-ADO structure (slate) and AAR$_{stearoyl\text{-}CoA}$-ADO structure (magenta). The stearoyl-CoA and residues involved in the interactions are shown as lines. In AAR$_{stearoyl\text{-}CoA}$ structure (magenta), the extended stearoyl-CoA (magenta) induces conformational change of the N-terminal region of AAR and helix 8 of ADO, and leads to the extra hydrogen bond formation between AAR and ADO. The hydrogen bonds are shown as black dashed lines.

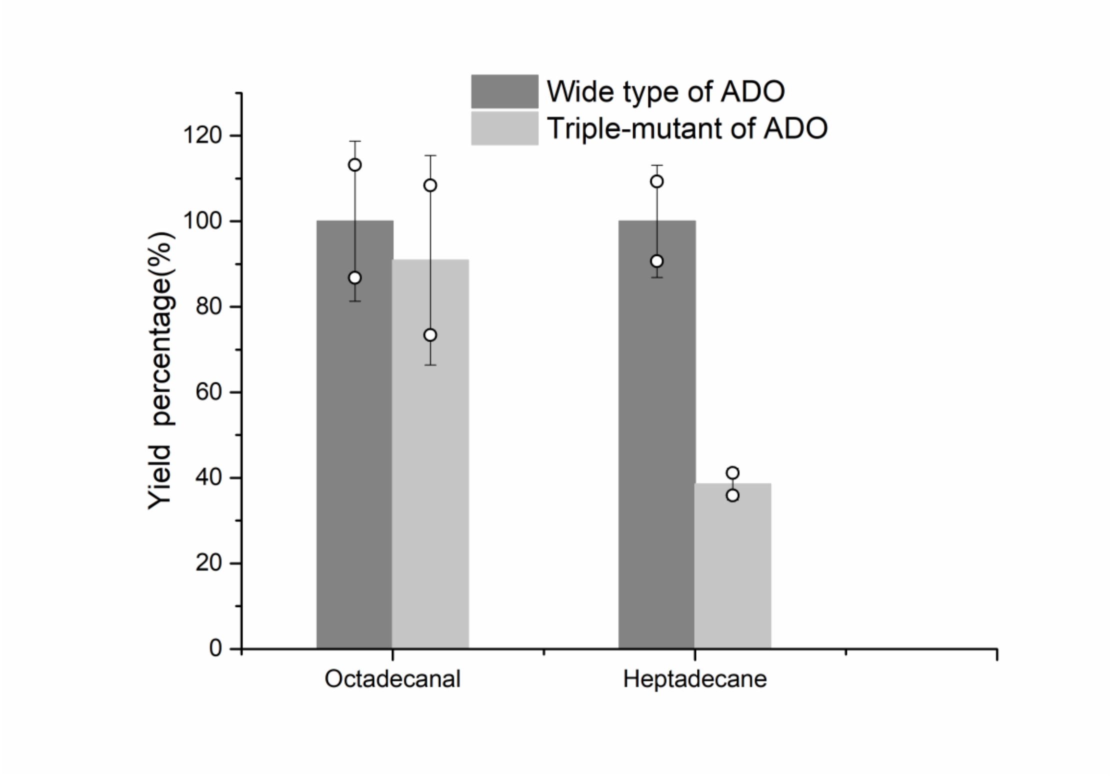

Supplementary Figure 13. Comparison of product yields of AAR incubated with either wild type or triple mutant of ADO. The products of AAR and ADO are octadecanal and heptadecane, respectively. The amount of octadecanal and heptadecane produced by wild type of AAR and ADO are normalized to 100% and shown in dark grey, while the amount of product produced by wild type and triple mutant of ADO are shown in light grey. The mean values and standard deviations were calculated from two independent measurements, with the corresponding data points shown as black circles. Source data are provided as a Source Data file.

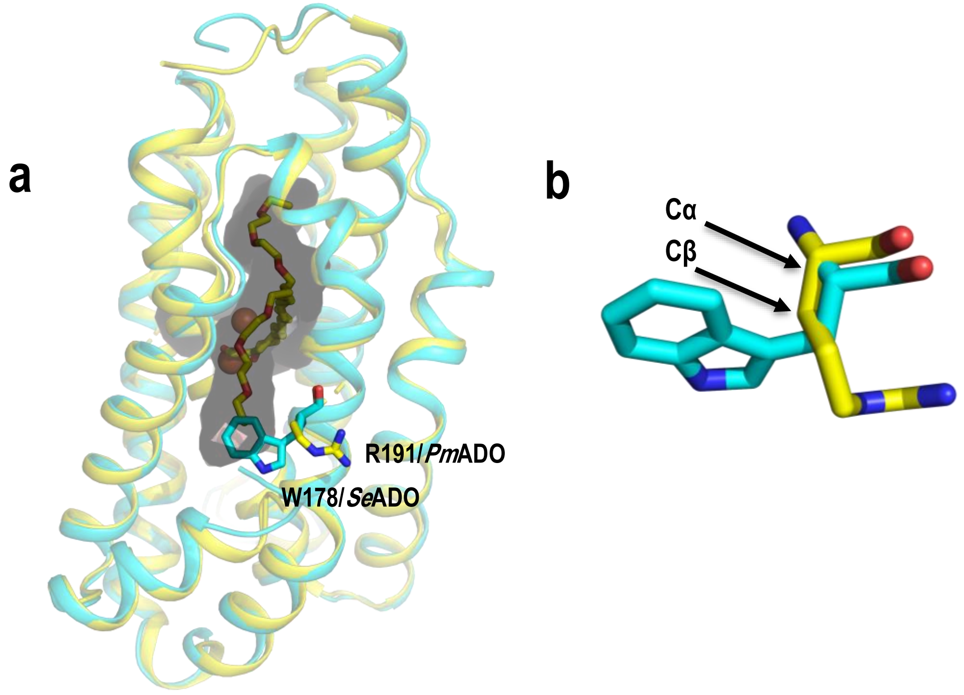

Supplementary Figure 14. Structural comparison of *Se*ADO and *Pm*ADO. (a) W178 in *Se*ADO corresponds to R191 in *Pm*ADO (PDB code 4PGI). The two residues are located at the entrance of ADO substrate tunnel and may function as a gate. *Pm*ADO and *Se*ADO are colored yellow and cyan, respectively. **(b)** The main chain atoms and C_β atoms of W178 in *Se*ADO (cyan) and R191 in *Pm*ADO (yellow) can be superposed well.

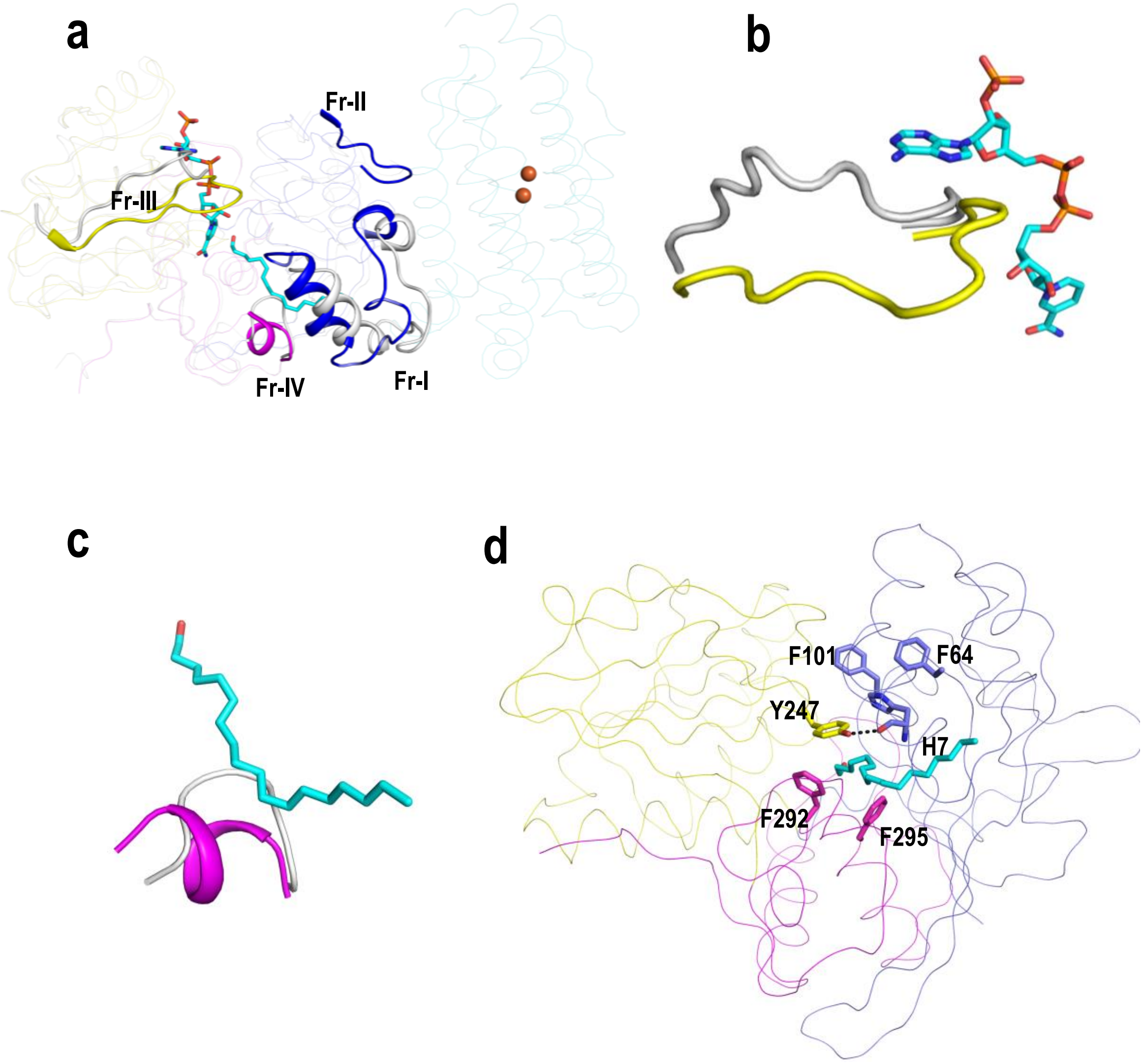

Supplementary Figure 15. Structure and comparison of AAR part in AAR$_{apo}$ and AAR-ADO complex structures. (a) Superposition of AAR$_{apo}$ and AAR-ADO complex (represented by AAR$_{NADPH}$-ADO) structures shown in ribbon mode. Three regions (Fr-1, Fr-III and Fr-IV) undergone drastic conformational changes and Fr-II involved in the AAR-ADO interactions are highlighted as cartoon mode. The NTD, mid-domain, CTD of AAR and ADO in AAR$_{NADPH}$-ADO structure are colored slate, yellow, magenta and cyan, respectively. AAR$_{apo}$ structure is colored white. NADPH and stearic chain bound with AAR are shown as sticks. Iron atoms bound with ADO are shown as brown spheres. **(b)** Enlarged view of the Fr-III region of AAR$_{apo}$ and AAR-ADO complex structures. NADPH is shown as sticks. **(c)** Enlarged view of the Fr-IV region of AAR$_{apo}$ and AAR-ADO complex structures. The stearic chain is shown as sticks. **(d)** The compact conformation of AAR in the AAR-ADO complex structure. Y247, H7 and the aromatic residues involved in the hydrophobic interactions are shown as sticks. The hydrogen bond between Y247 and H7 is shown as black dashed line.

Supplementary Table 1. DNA sequences of *Se*AAR and *Se*ADO

**DNA sequence**
***Se*AAR**
ATGTTCGGTCTTATCGGTCATCTCACCAGTTTGGAGCAGGCCCGCGACGTTTCTCGCAGGA<br>TGGGCTACGACGAATACGCCGATCAAGGATTGGAGTTTTGGAGTAGCGCTCCTCCTCAAA<br>TCGTTGATGAAATCACAGTCACCAGTGCCACAGGCAAGGTGATTCACGGTCGCTACATCG<br>AATCGTGTTTCTTGCCGGAAATGCTGGCGGCGCGCCGCTTCAAAACAGCCACGCGCAAAG<br>TTCTCAATGCCATGTCCCATGCCCAAAAACACGGCATCGACATCTCGGCCTTGGGGGGCT<br>TTACCTCGATTATTTTCGAGAATTTCGATTTGGCCAGTTTGCGGCAAGTGCGCGACACTAC<br>CTTGGAGTTTGAACGGTTCACCACCGGCAATACTCACACGGCCTACGTAATCTGTAGACA<br>GGTGGAAGCCGCTGCTAAAACGCTGGGCATCGACATTACCCAAGCGACAGTAGCGGTTGT<br>CGGCGCGACTGGCGATATCGGTAGCGCTGTCTGCCGCTGGCTCGACCTCAAACTGGGTGT<br>CGGTGATTTGATCCTGACGGCGCGCAATCAGGAGCGTTTGGATAACCTGCAGGCTGAACT<br>CGGCCGGGGCAAGATTCTGCCCTTGGAAGCCGCTCTGCCGGAAGCTGACTTTATCGTGTG<br>GGTCGCCAGTATGCCTCAGGGCGTAGTGATCGACCCAGCAACCCTGAAGCAACCCTGCGT<br>CCTAATCGACGGGGGCTACCCCAAAAACTTGGGCAGCAAAGTCCAAGGTGAGGGCATCT<br>ATGTCCTCAATGGCGGGGTAGTTGAACATTGCTTCGACATCGACTGGCAGATCATGTCCG<br>CTGCAGAGATGGCGCGGCCCGAGCGCCAGATGTTTGCCTGCTTTGCCGAGGCGATGCTCT<br>TGGAATTTGAAGGCTGGCATACTAACTTCTCCTGGGGCCGCAACCAAATCACGATCGAGA<br>AGATGGAAGCGATCGGTGAGGCATCGGTGCGCCACGGCTTCCAACCCTTGGCATTGGCAA<br>TT
***Se*ADO (optimized)**
ATGCCGCAGCTGGAGGCGTCTCTGGAACTGGATTTTCAGAGCGAGTCCTATAAAGACGCG<br>TACTCCCGCATCAACGCCATCGTGATTGAAGGTGAGCAGGAAGCATTTGATAACTATAAC<br>CGTCTGGCAGAAATGCTGCCGGATCAACGCGACGAACTGCATAAACTGGCGAAAATGGA<br>ACAGCGCCACATGAAAGGCTTCATGGCTTGCGGCAAGAATCTGAGCGTTACGCCAGATAT<br>GGGTTTCGCGCAAAAATTCTTCGAACGTCTGCACGAAAACTTTAAAGCCGCTGCTGCAGA<br>AGGTAAAGTTGTGACCTGTCTGCTGATCCAGTCTCTGATTATCGAGTGCTTCGCTATCGCT<br>GCATACAACATCTACATCCCGGTCGCTGATGCGTTCGCACGTAAAATCACCGAAGGTGTG<br>GTTCGTGACGAATACCTGCACCGTAACTTCGGCGAAGAATGGCTGAAAGCGAATTTCGAT<br>GCTTCTAAGGCAGAGCTGGAAGAGGCGAACCGTCAGAACCTGCCTCTGGTATGGCTGATG<br>CTGAACGAAGTTGCAGACGACGCTCGCGAACTGGGCATGGAACGTGAATCTCTGGTTGAA<br>GACTTCATGATTGCCTACGGCGAAGCCCTGGAAAACATCGGTTTTACCACTCGTGAGATT<br>ATGCGTATGTCCGCCTATGGCCTTGCGGCCGTT

Supplementary Table 2. Primer sequences

		Primer sequences
**AAR**		
WT	U	5'GGGAATTCCATATGTTCGGTCTTATCGGTCAT3'
	L	5'GGAATTCATGCTGCCCTGGAAATACAGAT TTTCAATTGCCAATGCCAAGGGTTG3'
R73A	U	5' GCGGCGCGCGCATTCAAAACAGCCACGCGCAAAGTT 3'
	L	5' GGCTGTTTTGAAGCGTGCCGCCGCCAGCATTTCCGGCAA 3'
R79A	U	5' ACAGCCACGGCAAAAGTTCTCAATGCCATGTCCCAT 3'
	L	5' ATTGAGAACTTTTGCCGTGGCTGTTTTGAAGCGGC 3'
K80A	U	5' ACAGCCACGCGCGCAGTTCTCAATGCCATGTCCCAT 3'
	L	5' ATTGAGAACTGCGCGCGTGGCTGTTTTGAAGCGG 3'
C294S	U	5'GAGCGCCAGATGTTTGCCAGCTTTGCCGA3'
	L	5'TCGGCAAAGCTGGCAAACATCTGGCGCTC3'
Y247F	U	5'ATCGACGGGGGCTTTCCCAAAAACTTG3'
	L	5'CAAGTTTTTGGGAAAGCCCCCGTCGATTA3'
Y247A	U	5'CTAATCGACGGGGGCGCTCCCAAAAACTTG3'
	L	5'CAAGTTTTTGGGAGCGCCCCCGTCGAT3'
**ADO**		
WT	U	5'CATATGCCGCAGCTTGAAGCCAGCCT3'
	L	5'CGGGATCCATGCTGCCCTGGAAATACAGATTTTCAACGGCCGC AAGGCCATAGGCGG3'
E196A	U	5' ATGGAACGTGCATCTCTGGTTGAAGACTTC 3'
	L	5' AACCAGAGATGCACGTTCCATGCCCAGTTC 3'
E200A	U	5' TCTCTGGTTGCAGACTTCATGATTGCCTAC 3'
	L	5' CATGAAGTCTGCAACCAGAGATTCACGTTC 3'
D201A	U	5' CTGGTTGAAGCATTCATGATTGCCTACGGC 3'
	L	5' AATCATGAATGCTTCAACCAGAGATTCACG 3'
208A	U	5' GCCTACGGCGCAGCCCTGGAAAACATC 3'
	L	5' TTCCAGGGCTGCGCCGTAGGCAATCAT3'
E211A	U	5'GCCTACGGCGAAGCCCTGGCAAACATCGGTTT3'
	L	5'CGAGTGGTAAAACCGATGTTTGCCAGGGCTTC3'
E200A\D201A	U	5'GAACGTGAATCTCTGGTTGCAGCATTCATGAT3'
	L	5'CCGTAGGCAATCATGAATGCTGCAACCAGAGA3'
E196A\E200A\D 201A	U	5'CTGGGCATGGAACGTGCATCTCTGGTTGCAGCATTCAT3'
	L	5'GTAGGCAATCATGAATGCTGCAACCAGAGATGCACGTTC3'

* “U” and “L” represent upper primer and lower primer respectively.
